# Extrinsic Factors Involved in the Differentiation of Stem Cells into Insulin-Producing Cells: An Overview

**DOI:** 10.1155/2011/406182

**Published:** 2011-06-16

**Authors:** Rebecca S. Y. Wong

**Affiliations:** Division of Human Biology, School of Medical and Health Sciences, International Medical University, No. 126, Jalan Jalil Perkasa 19, Bukit Jalil, 57000 Kuala Lumpur, Malaysia

## Abstract

Diabetes mellitus is a chronic disease with many debilitating complications. Treatment of diabetes mellitus mainly revolves around conventional oral hypoglycaemic agents and insulin replacement therapy. Recently, scientists have turned their attention to the generation of insulin-producing cells (IPCs) from stem cells of various sources. To date, many types of stem cells of human and animal origins have been successfully turned into IPCs *in vitro* and have been shown to exert glucose-lowering effect *in vivo*. However, scientists are still faced with the challenge of producing a sufficient number of IPCs that can in turn produce sufficient insulin for clinical use. A careful choice of stem cells, methods, and extrinsic factors for induction may all be contributing factors to successful production of functional beta-islet like IPCs. It is also important that the mechanism of differentiation and mechanism by which IPCs correct hyperglycaemia are carefully studied before they are used in human subjects.

## 1. Introduction

Diabetes mellitus is a common disease in many parts of the world with serious complications and no cure. Since the discovery of insulin more than 80 years ago, the treatment of diabetes mellitus has not changed much. Although cadaveric pancreatic transplantations have been explored, they are not without disadvantages and complications such as difficulty in finding a suitable donor and immune rejection of the transplanted pancreas or islet cells [1, 2]. This has led researchers to venture into alternative ways of treating diabetes mellitus. The ability of stem cells of various origins to differentiate into IPCs has brought new therapeutic hopes to sufferers of the disease [3, 4]. The sources of stem cells used in the generation of IPCs have been previously reviewed [5–7]. However, the literature on the methodology and extrinsic factors involved in differentiation of stem cells into IPCs is scarce. This article gives an overview of sources of IPCs with an emphasis on various methods and extrinsic factors used and the potential problems and practical values of such *in vitro* differentiation. 

## 2. The Pancreas and Its Development

 The pancreas is a gland organ that plays an important role in the digestive and endocrine systems. It consists of two major types of cells: (1) exocrine cells, which are organized in acini and secrete digestive enzymes and (2) endocrine cells, which are organized in the islets of Langerhans and secrete hormones into the bloodstream. In an adult pancreas, the islets contribute to 1% to 2% of the total pancreatic mass. There are approximately one million islets with three main types of cells, namely, alpha, beta, and delta cells. The insulin-secreting beta cells, and glucagon-secreting alpha cells contribute to 75% and 25% of the islets cells, respectively, while the remaining 5% of islet cells is made up of the somatostatin-secreting delta cells. Another type of cells, called the F cells (or PP cells), secretes pancreatic polypeptide. These cells are present mainly in the islets situated in the posterior portion of the head of pancreas, and the physiological function of pancreatic polypeptide is largely unknown [8].

 The mature pancreas is a single organ, which is initially derived from two separate and distinct rudiments, that is a dorsal and a ventral bud arising from the primitive gut epithelium. The dorsal pancreatic bud grows into the dorsal mesentery while the ventral bud, the ventral mesentery. The differential rotation and fusion of the dorsal and ventral pancreatic buds, which take place late in the sixth week of fetal development, result in the formation of the definitive organ and are followed by the interconnection of their ductal systems [9]. The ventral bud gives rise to the uncinate process and the inferior part of the head of the pancreas whereas the dorsal bud forms the remaining part of the gland. The distal part of the dorsal pancreatic duct and the entire ventral pancreatic duct form the main pancreatic duct (of Wirsung), which together with the bile duct, enters the duodenum at the major papilla [10]. The islets of Langerhan develop from the pancreatic parenchymal tissue during the third month of life and are scattered throughout the pancreas. During the fifth month of fetal development, insulin-secretion begins while glucagon- and somatostatin-secreting cells also develop from parenchymal cells [10]. The coexpression of several hormones by the same cell is often seen in the early stage of pancreatic development [11]. 

 The expression of the pancreatic and duodenum homeobox-1 (Pdx1) gene, also known as insulin promoter factor-1 (Ipf1), has been shown to be important in pancreatic development, and beta-cell maturation and inactivity leads to total absence of the organ [12]. Other factors which have been shown to play a role in the development of the pancreas and/or the differentiation of insulin-producing beta cells include Pax4 [13], NeuroD/Beta2 [14], epidermal growth factor (EGF) [15], transforming growth factor-beta (TGF-*β*), prospero-related homeobox transcription factor-1(Prox1), NKx transcription factors, and neurogenin-3 (Ngn3) [16]. 

## 3. Glucose Homeostasis

 Although many hormones are capable of increasing blood sugar levels, insulin is the only hormone that has a direct glucose-lowering effect. It is one of the key regulators of blood glucose concentration, and it plays an important role in the pathophysiology of diabetes mellitus. Insulin's main actions on carbohydrate metabolism include (1) increasing glucose uptake in target cells, hence promoting storage in the form of glycogen, (2) stimulation of glycogenesis, (3) inhibition of gluconeogenesis, and (4) decreasing hepatic glucose output by inhibiting gluconeogenesis. Together, these actions contribute to insulin's glucose-lowering effect. In addition, insulin also exerts multiple effects in fat and protein metabolisms [17–20]. The control of blood glucose concentration is a complex process. Briefly, when blood glucose concentration is raised, the pancreatic beta cells are stimulated to increase insulin secretion, which results in a decrease in blood glucose concentration, accompanied by a decrease in blood fatty acid and amino acid concentrations. Besides, there is also an increase in protein synthesis and fuel storage. Other factors that stimulate beta-cell insulin secretion include parasympathetic stimulation following food intake, an increase in blood amino acid and free fatty acid concentrations, intestinal hormones such as gastrin, cholecystokinin, secretin, glucagon-like peptide-1, and glucose-dependent insulinotropic polypeptide (previously known as gastric inhibitory polypeptide) [17, 19, 20]. The gut-derived hormones are said to have an incretin effect, which means they increase insulin release after an oral nutrient load [21]. On the other hand, a decrease in blood glucose concentration, sympathetic stimulation, somatostatin, and leptin inhibis, beta-cell insulin secretion [17, 20]. Besides insulin, glucagon is another hormone that plays a key role in glucose homeostasis, and its actions generally oppose those of insulin such as the promotion of glycogenolysis, gluconeogenesis, and the breakdown of fats. It also increases amino acid uptake by liver cells, which in turn increases the conversion of amino acids to glucose in the liver [19]. The actions of insulin and glucagon and factors that affect their secretion are summarised in Table 1. 

## 4. Diabetes Mellitus as a Global Health Problem

Diabetes mellitus is a group of metabolic diseases with hyperglycaemia as a hallmark. It is due to defects in insulin secretion or action, or both. Chronic hyperglycaemia is associated with many debilitating complications involving multiple organs such as the eyes, kidneys, heart, nerves, and blood vessels [22]. Most cases of diabetes mellitus can be divided into two main categories according to its pathogenesis, namely, type 1 diabetes mellitus (T1DM) and type 2 diabetes mellitus (T2DM). The former comprises 5–10% of all DM cases and the latter, 90–95% of all DM cases [22]. The pathogenic processes underlying the disease are complex. T1DM results from a cellular-mediated autoimmune destruction of the beta-cells of the pancreas leading to absolute insulin deficiency while T2DM is caused by a combination of peripheral resistance to insulin action and relative insulin deficiency due to an inadequate response in insulin secretion [23]. 

 The number of people with diabetes mellitus is ever increasing. Some contributing factors to this increase include population growth, aging, urbanisation, increasing prevalence of obesity and physical inactivity [24]. Wild et al. reported that the worldwide prevalence for diabetes mellitus for all ages was estimated to be 2.8% in 2000 and 4.4% in 2030. The total number of people with diabetes mellitus is estimated to increase from 171 million in 2000 to 366 million in 2030 even if the prevalence of obesity remains stable till 2030 [24]. Such increase in the prevalence of diabetes is alarming as it will also contribute to an increase in deaths as a result of cardiovascular and other diabetic complications. Therefore, urgent global attention to address these issues is necessary.

 The treatment of diabetes mellitus mainly revolves around oral hypoglycaemic drugs and insulin replacement therapy. Pancreatic islets transplantation is an alternative, but its use is restricted by a shortage of donated organs, immune rejection, and the need of life-long immunosuppression [1, 2]. Therefore, IPCs generated from stem cells may represent a new therapeutic alternative and a potential source for beta cell replacement. 

## 5. Sources of Insulin-Producing Cells

To date, many different types of human or animal stem/progenitor cells have been used for the generation of IPCs *in vitro*. Potential sources of IPCs can generally be divided into 3 main categories: (1) embryonic stem cells, (2) adult stem/progenitor cells of pancreatic origin, and (3) adult stem/progenitor cells of nonpancreatic origins. 

### 5.1. Embryonic Stem Cells

 Embryonic stem cells (ESCs) are defined as self-renewing pluripotent cells derived from the inner cell mass of blastocysts. ESCs are unique in that they can be indefinitely cultured in an undifferentiated state and be differentiated into cells of the three embryonic germ layers, namely, the ectoderm, mesoderm, and endoderm [25, 26]. Thus far, ESCs from both animal and human origins have been shown to differentiate into IPCs [3, 4, 27]. Several arguments have long existed with the use of ESCs, especially if they are of human origin. The enormous proliferative and differentiation capacity of embryonic stem cells raises the concern of the development of teratoma [28]. The use of ESCs for the generation of IPCs may require therapeutic cloning of human ESCs which may in turn leads to many ethical issues. 

### 5.2. Adult Stem/Progenitor Cells of Pancreatic Origin

 Adult stem cells differ from embryonic stem cells in that they are restricted to differentiate into a variety of cell types with a defined lineage. Therefore, differentiation of stem cells into cells of a different lineage is considered a form of transdifferentiation. Several studies have looked into such transdifferentiation of adult stem cells into IPCs using adult stem cells of both pancreatic and non-pancreatic origins.

 For many years, scientists were misled to think that beta cells did not replicate based on the commonly accepted concept that terminally differentiated cells do not replicate. It is now clear that new beta cells are formed in the adult pancreas [29]. Hence, the pancreas may represent a potential source of various types of stem or progenitor cells for the generation of IPCs. So far, stem or progenitor cells that have been differentiated into IPCs were derived from (1) pancreatic islets [30], (2) pancreatic ducts [31], (3) pancreatic acinar cells [32], and (4) within adult or fetal pancreas without further specification [33]. 

### 5.3. Adult Stem/Progenitor Cells of Non-Pancreatic Origins

**Figure 1:**
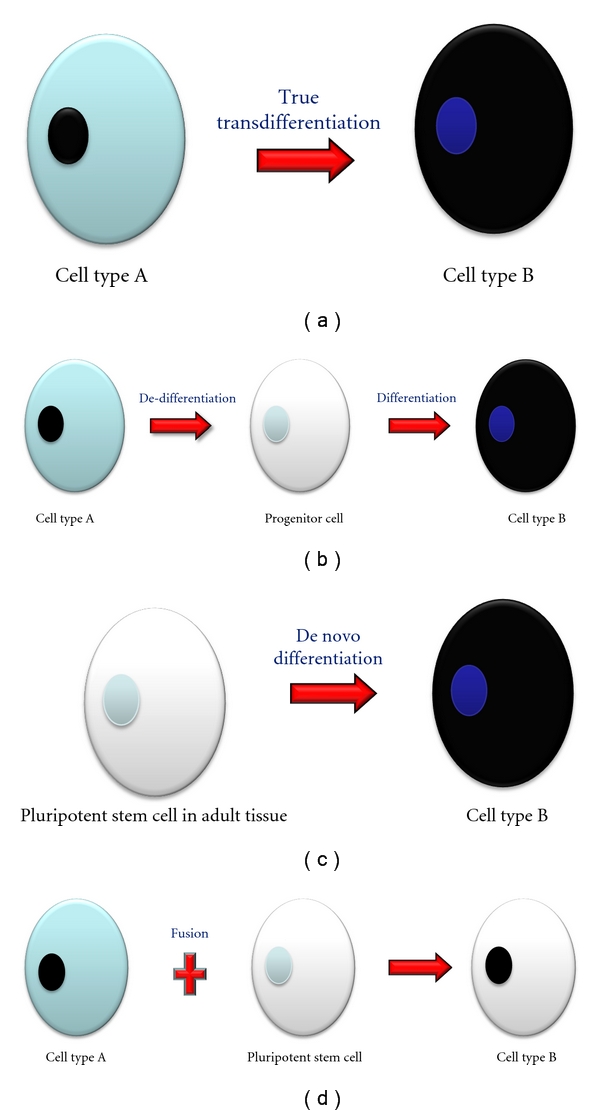
Possible pathways of generation of differentiated cells from another cell type. (a) True differentiation of one cell type into another. (b) De-differentiation of one cell type into a common progenitor cell followed by differentiation into another cell type. (c) De novo differentiation of a pluripotent stem cell in adult tissue into another cell type. (d) Fusion of one cell type with a pluripotent stem cell giving rise to another cell type.

 Besides stem or progenitor cells of pancreatic origin, other adult stem cells of both animal and human origins have been shown to transdifferentiate into IPCs. The possible pathways for the generation of differentiated cells from a different tissue include (1) true transdifferentiation of a differentiated cell into another differentiated cell, (2) de-differentiation of a differentiated cell into a common progenitor cell type, followed by differentiation into another cell type which is different from that of the original cell, (3) de novo differentiation of pluripotent cells which have persisted in adult tissues, and (4) fusion of a pluripotent cell with another already differentiated cell (Figure 1) [34]. The list of adult stem cells used in such transdifferentiation is exhaustive. Among these adult stem cells, mesenchymal stem cells (MSCs) have been widely used as a source of IPCs in the literature. Sources of MSCs which have been shown to have successfully transdifferentiated into IPCs include the bone marrow [35, 36], umbilical cord blood [37], and adipose tissue [38]. MSCs have the advantage over ESCs in that they usually do not form teratomas and are free from the ethical issues of ESCs. These cells can be easily obtained and are easily expanded and cultured in the laboratory. Other organs such as the liver [39, 40], spleen [41], intestine [42], and brain [43] have also been shown to be sources of IPCs. 

## 6. Methods and Extrinsic Factors Used in the Generation of Insulin-Producing Cells

The vast variety of stem cells used makes comparison of methods difficult. Different types of stem cells require different culture and induction media for differentiation of IPCs to take place. However, some common themes seem to appear in various induction methods.

Firstly, induction of stem or progenitor cells into IPCs usually requires a multistage protocol. Most protocols require at least a two-stage protocol [44] with others up to five [27, 52] or six stages [3]. It is observed that induction of ESCs generally requires more stages compared to stem cells of other origins [3, 52, 61].

Secondly, the induction period varies greatly with the type of cells used. It may last from several days to several months. For example, in a study carried out by Chen et al., the protocol involved a two-stage protocol during which rat bone marrow mesenchymal stem cells were preinduced for 24 hours and reinduced for an additional 10 hours. The entire induction process took less than 48 hours [44]. On the other hand, in another study done by Tang et al. using mouse bone marrow-derived stem cells, clusters of IPCs started to appear in two months with better-defined clusters appearing in four months [45]. Such wide variations in the induction period are not uncommon in published literature. A summary of various induction protocols is given in Table 2.

Lastly, addition and withdrawal of a combination of extrinsic factors in a stage-wise manner are common to most protocols. Many extrinsic factors have been shown to promote beta-cell proliferation and differentiation and increase insulin content of IPCs. A number of these factors have been commonly observed in induction protocols. Careful use of serum and glucose in the induction media has also been indicated for successful generation of IPCs. A summary of these extrinsic factors and their effects is given in Table 3. 

### 6.1. Epidermal Growth Factor (EGF) and Basic Fibroblast Growth Factor (bFGF)

EGF is a growth factor belonging to the EGF family of proteins. It plays an important role in cellular proliferation, differentiation, and survival [62]. EGF acts by binding to epidermal growth factor receptor (EGFR) on the cell surface which stimulates the intrinsic protein-tyrosine kinase activity of the receptor leading to the initiation of a signal transduction cascade. This in turn leads to a series of biochemical changes in the cell such as an increase in intracellular calcium levels, increased glycolysis and protein synthesis, and expression of certain genes, all of which lead to DNA synthesis and cell proliferation [63]. 

Fibroblast growth factor (FGF) was first discovered as an activity in extracts of pituitary and brain which had a stimulatory effect on the growth of mouse fibroblast cells. It was later shown that the activity was due to two proteins, namely, acidic fibroblast growth factor (aFGF) and basic fibroblast growth factor (bFGF) [64]. bFGF is also known as FGF2 or FGF-*β*, which is a member of the fibroblast growth factor family of proteins. bFGF demonstrates a broad spectrum of biological activities ranging from increased growth and cell migration on many cell types *in vitro* to involvement in neovascularization and wound repair *in vivo* [64]. 

 Low concentrations of EGF and bFGF are used in the culture of stem cells, especially the MSCs. Interestingly, high concentrations of EGF and bFGF, either used alone, or in combination, have been shown to be useful in IPC differentiation [35, 52]. On the contrary, Dalvi et al. demonstrated that when human pancreatic islets were exposed to medium containing a high concentration of EGF (50 ng/mL), such populations demonstrated a high degree of proliferation [49]. Removal of EGF from the medium resulted in the formation of islet-like cell aggregates. This phenomenon was also supported by Cras-Méneur et al. who demonstrated that EGF increased undifferentiated pancreatic embryonic cells *in vitro*. Therefore, a careful use of EGF may be important in successful IPC differentiation [51]. It is also important to note that bFGF may play a role in the clustering of IPCs. In a study performed by Hardikar et al., it was shown that out of several growth and differentiation factors tested on pancreatic duct progenitor cells, bFGF secreted by endocrine precursor cells was found to be the most effective chemoattractant in the clustering of these pancreatic precursor cells [50]. 

### 6.2. Activin A and Betacellulin

 Activin proteins are members of the transforming growth factor-beta (TGF-*β*) superfamily. Activin A has a wide range of biological activities including regulation of cellular proliferation and differentiation and promotion of neuronal survival. Like other members of the TGF-*β* superfamily, activin A has been reported to affect embryogenesis, haematopoiesis and angiogenesis [65–67]. Activin A is found in abundance in bone matrix [68], and bone marrow-derived fibroblasts are a major source of activin A [69]. 

Betacellulin is a member of the EGF family isolated as a 32 kDa glycoprotein from the conditioned medium of a mouse pancreatic insulinoma cell line [70]. It is widely expressed in many organs and highly expressed in the pancreas and intestine [71]. It is also expressed in human foetal pancreas [72]. Much of the knowledge about the biological function of this protein mainly came from studies of its effect on cultured cell line *in vitro*. In a study carried out by Demeterco et al. to analyze the effect of activin A and betacellulin on islet development and growth, it was found that betacellulin acted as a mitogen for undifferentiated pancreatic epithelial cells with an increase in the number of islet-like clusters. On the other hand, activin A was responsible for increased insulin contents. Interestingly, combined use of both factors led to weaker effects when compared to the use of either of the factor alone [53]. It has also been shown that intraperitoneal injection of betacellulin enhanced beta cell regeneration in 90% pancreaticised rats [73]. 

 The use of betacellulin and activin A, either alone or in combination, has resulted in differentiation of stem cells into IPCs [35, 40, 54]. For example, Sun et al. showed that combined use of betacellulin and activin A was involved in the differentiation of bone marrow-derived mesenchymal stem cells from diabetic patients into IPCs [35]. On the other hand, Thowfeequ et al. demonstrated that betacellulin inhibited amylase and glucagon production and promoted beta cell differentiation in mouse embryonic pancreas [74]. Using betacellulin and/or activin A, other studies have successfully differentiated stem cells of liver [40] and pancreatic [54] origins into IPCs. 

### 6.3. Nicotinamide

Nicotinamide is also called niacinamide or nicotinic acid amide. It is the amide of nicotinic acid or vitamin B3. Nicotinamide represents another commonly used extrinsic induction factor. As early as the 1990s Otonkoski et al. had used nicotinamide as an inducer of endocrine differentiation in cultured human foetal pancreatic cells [55]. It was shown that treatment of human foetal pancreatic cells with 10 nM nicotinamide resulted in a twofold increase in DNA content and a threefold increase in insulin content associated with development of beta cell outgrowths from undifferentiated epithelial cell clusters. There was also an increase in the expression of the insulin, glucagon, and somatostatin genes [55]. More recently, many studies almost inevitably used nicotinamide in their induction protocols in one or more stages together with other extrinsic induction factors [3, 35–37, 44–48]. 

### 6.4. Exendin-4

 Exendin-4 is a 39 amino acid protein isolated from the venom of the lizard *Heloderma suspectum *[75]. Several studies have been carried out to elucidate the effect of exendin-4 *in vivo*. Xu et al. demonstrated that exendin-4 increased beta cell mass by stimulating beta cell proliferation and neogenesis in diabetic rats [76] while Yang et al. demonstrated that when combined with lisofylline, treatment with exendin-4 reversed pancreatic beta cell destruction in diabetic rats [77]. Exendin-4 has been shown to differentiate stem cells of various origins into IPCs [36, 37, 45, 47, 56]. It has also been reported that high levels of the protein *in vitro* resulted in an increase in insulin release by IPCs derived from mouse embryonic stem cells [56]. 

### 6.5. Hepatocyte Growth Factor (HGF)

HGF is a heparin-binding protein secreted by mesenchymal cells. It is an inducer of cell proliferation, cell motility, and morphogenesis in many cell types. HGF has been reported to stimulate T cell adhesion to endothelium and migration as well as to enhance neuron survival. It has also been found to play a role in the regulation of erythroid differentiation and has an inhibitory effect on cell growth [78]. Several studies have used HGF as one of the extrinsic factors in IPC differentiation [40, 54, 57]. It was shown that with further treatment of HGF, activin A-treated AR42J cells differentiated into IPCs [54]. HGF was also shown to increase the number of IPCs in cultured human islets by Otonkoski et al. [57]. 

### 6.6. Gastrin

Gastrin is a hormone produced by specialised cells called G cells in the antral part of gastric mucosa. Small amounts of gastrin are produced by the duodenum and the pancreas. It is also found in the pancreatic islets in foetal life. Its principle physiologic role is to stimulate gastric acid and pepsin secretion and to stimulate growth of the mucosa of the stomach and small and large intestine [79].Wang et al. demonstrated that gastrin stimulated islet differentiation of TGF-*α* induced ductular precursor cells [58]. Gastrin and TGF- *α* were found to be expressed during duct to islet cell differentiation in the pancreas of duct-ligated adult rats [59]. On the other hand, Tamaki et al. reported that combination treatment of exendin-4 and gastrin preserves beta cell mass by stimulating beta cell growth and differentiation in *db/db* mice [80]. 

### 6.7. Glucose and Serum

Interestingly, the concentration of glucose in the induction medium seemed to play a role in IPC differentiation. Bonner-Weir et al. demonstrated that low concentrations of glucose (5 mM) increased insulin content in islet-like clusters while higher concentrations (20–30 mM) increased beta cell replication *in vivo* and *in vitro*. Therefore, it is not uncommon that an induction protocol varies the glucose concentration of its induction medium from one stage to another to promote optimal beta cell replication, and insulin content and secretion [60]. Besides, the presence or absence of serum in the induction medium appeared to have an effect on IPC differentiation. Some protocols have a serum-free stage, usually early in induction [35, 36, 44]. It was demonstrated that withdrawal of foetal bovine serum from culture medium encouraged differentiation of islet-like clusters that secrete insulin upon glucose stimulation [81]. 

## 7. Challenges Faced in Differentiation of Stem Cells to IPCs

Although several studies have demonstrated stem cells of various origins can be differentiated into IPCs, the small amounts of insulin secreted by these IPCs *in vitro* may not be very useful clinically. The wide variations in the type of stem cells used, whether these cells were from a human or animal source, the initial number of cells used in induction, the number of clusters formed and the induction method used are all factors that need to be taken into consideration, which makes comparison and standardisation difficult. 

 Some of the challenges faced by researchers include (1) the choice of cell and an induction method which can consistently produce functional, beta islet-like IPCs and (2) generation of IPCs that can consistently produce clinically significant amounts of insulin. Although several studies have demonstrated that IPCs generated *in vitro* could correct hyperglycaemia in animal models [27, 36, 40, 43–45, 47, 61], it is not certain whether differentiated cells can correct hyperglycaemia in human subjects. The long-term fate of these IPCs injected into animals is also unknown. Areas that require exploration include the mechanism involved in differentiation of various stem cells into IPCs, the mechanism by which IPCs improve hyperglycaemia, and the fate of the injected IPCs in animal models. 

## 8. Conclusions and Future Perspectives

Stem cells of various origins may be an important source of IPCs. The abundance of literature suggests that the generation of stem cells into IPCs is feasible and promising. However, the wide variations in induction techniques and sources of stem cell used may be a challenge to researchers as there is no standard method for IPC generation. A careful choice of stem cell and induction method is necessary for successful IPC differentiation. Further exploration is necessary for the generation of sufficient IPCs that can produce sufficient insulin for clinical use and the mechanism of such differentiation and how these differentiated cells correct hyperglycaemia need to be established before they can be used in human subjects for the treatment of diabetes mellitus. 

##  Conflict of Interests

The author declares that there is no conflict of interests. 

## Figures and Tables

**Table 1:** Actions of insulin and glucagon and factors that affect their secretion.

Hormone	Action	Factors affecting secretion
Insulin	*Carbohydrate metabolism*	*Increased secretion*
	Increases glucose uptake into target cells (e.g., skeletal and	Increased blood glucose concentration
	adipose tissue cells)	
	Stimulates glycogenesis in skeletal muscle and liver	Parasympathetic stimulation following food intake
	Inhibits gluconeogenesis	Increased blood amino acid concentration
	Decreases hepatic output of glucose by	Increased free fatty acid concentration
	inhibiting.gluconeogenesis	
	*Fat metabolism*	Intestinal hormones (e.g., gastrin, cholecystokinin,
		secretin, glucagons-like peptide 1, and glucose-.
		dependent insulinotropic polypeptide)
	Increases fatty acids and triglyceride synthesis by liver	Glucagon
	Increases entry of fatty acids from blood into adipose.	Growth hormone
	tissues	
	Inhibits lipolysis, decreasing release of fatty acids from	Cortisol
	adipose tissue	
	*Protein metabolism*	Insulin resistance
	Increases active transport of amino acids into target cells	Obesity
	(e.g., muscle cells)	
	Increases protein synthesis	*Decreased secretion*
	Inhibits protein catabolism	Decreased blood glucose concentration
		Fasting
		Sympathetic stimulation
		Somatostatin
		Leptin

Glucagon	*Carbohydrate metabolism*	*Increased secretion*
	Increases glycogenolysis	Decreased blood glucose concentration
	Increases gluconeogenesis	Increased blood amino acid concentration
	*Fat metabolism*	Increased catecholamines
	Increases lipolysis, making increased amounts of fatty acids available to the body	Sympathetic stimulation
		Exercise
	*Protein metabolism*	*Decreased secretion*
	Increases amino acid uptake by liver cells	Increased blood glucose concentration
	Increases conversion of amino acid to glucose by	Increased blood free fatty acid concentration
	gluconeogenesis in the liver	
		Somatostatin
		Insulin

**Table 2:** Summary of induction protocols used in insulin-producing cell generation.

Summary of protocol	Stem cell used in induction	Duration of induction	Remarks	Author
Stage 1: culture of undifferentiated human embryonic stem cells (hES)—DMEM, 20% knockout serum replacement, glutamine, nonessential amino acid, *β*-mercaptoethanol, and bFGF. Cells were dissociated after 30 minutes		Stage 1: Cells were dissociated after 30 minutes	Differentiated cells showed enhanced expression of pancreatic genes. Immunofluorescence and *in situ* hybridization showed a high percentage of insulin-producing cells in clusters, with most cells co-expressing somatostatin or glucagons, resembling immature pancreatic cells.	
Stage 2: generation of embryoid bodies 80% knockout DMEM, 20% FBS, glutamine, and non-essential amino acids		Stage 2: 7 days		
Stage 3: culture of embryoid bodies in DMEM/F12 medium with insulin-transferrin-selenium-fibronectin		Stage 3: 7 days		Segev et al., 2004 [3]
Stage 4: Expansion of pancreatic progenitor cells in DMEM/F12 medium with N2 & B27 supplement, bFGF	Human embryonic stem cells	Stage 4: 7 days		
Stage 5: withdrawal of bFGF, addition of nicotinamide, and reduction of glucose concentration		Stage 5: 4 days		
Stage 6: formation of clusters in suspension		Stage 6: cluster formation		
		Total: 25 days or longer		

Stage 1: Preinduction in L-DMEM medium with *β*-mercaptoethanol and nicotinamide	Rat marrow mesenchymal stem cells	Stage 1: 24 hours	Islet-like clusters were observed showing positive insulin mRNA and protein expressions. Differentiated cells responded to glucose challenge *in vitro* and could downregulate glucose in streptozotocin-induced diabetic rats.	Chen et al., 2004 [44]
Stage 2: Reinduction in serum-free HDMEM medium with nicotinamide, *β*-mercaptoethanol		Stage 2: 10 hours		
		Total: 34 hours		

Stage 1: RPMI medium, 10% FCS	Murine bone marrow-derived cells	Stage 1: 2 to 4 months	Differentiated cells expressed multiple genes related to pancreatic beta cell development and function. Insulin and C-peptide production was confirmed by immunocytochemistry and electron microscopy.* In vitro* insulin release was glucose stimulated.	
Stage 2: RPMI medium, glucose, 5% FCS, and nicotinamide		Stage 2: 7 days		Tang et al., 2004 [45]
Stage 3: RPMI medium, 5% FCS, glucose, nicotinamide, and exandin-4		Stage 3: 5–7 days	Transplantation of differentiated cells showed reversal of hyperglycaemia in streptozotocin-induced diabetic mice.	
		Total: variable		

H-DMEM serum-free medium, insulin, transferring, selenium, activin A, betacellulin, exendin-4, and hepatocyte growth factor	Human liver-derived fetal cells (FH-B-TPN)	Manipulation of culture conditions in various experimental settings	Cells cultured with activin A and betacellulin serum-free medium showed upregulation of NeuroD, Nkx22, glucokinase, prohormone convertase 1/3 and downregulation of Pax6, pancreatic polypeptide, glucagon, and liver markers. Insulin content of cultured cells increased 33-fold that of normal beta cells. *In vitro* insulin release responded to physiological glucose levels. Transplanted cultured cells in diabetic mice resulted in restoration of stable euglycaemia with continued *in vivo* insulin expression and no cell replication	Zalzman et al., 2005 [40].

Stage 1: neurosphere cell line cultured in expanded in medium with X-VIVO15, N2 supplement, heparin, leukaemia inhibitory factor, EGF, and bFGF	Human neurospheres cell lines	Stage 1: cells were frozen after expansion	Formation of glucose-responsive, insulin-producing cells in clusters. Expression of various genes involved in pancreatic development	
Stage 2: DMEM/F12 medium with, bovine serum albumin, N2 supplement, heparin, leukaemia inhibitory factor, EGF, and bFGF		Stage 2: 14 days		
Stage 3: L-DMEM/F12 medium, apo-transferrin, glucose, bovine insulin, sodium selenite, and retinoic acid		Stage 3: 14 days	Transplantation of differentiated cells into immunocompromised mice showed release of insulin C-peptide upon glucose challenge transplanted cells did not differentiate further and did not form tumours.	Hori et al., 2005 [43]
Stage 4: N2 medium, nicotinamide, insulin-like growth factor-1, and glucose		Stage 4: 6 days		
		Total: 34 days (excluding cell expansion in stage 1)		

Serum free DMEM/F12 medium, glucose, nicotinamide, activin-A, exendin-4, hepatocyte growth factor, pentagastrin, B27 supplement, and N2 supplement	Human adipose-derived mesenchymal stem cells	Gene expression profile was analyzed every 24 hours for 3 days.	Down-regulation of ABCG-2 and up-regulation of pancreatic developmental transcription factors (Isl-1, Ipf-1 and Ngn3) were observed, together with induction of islet hormones insulin, glucagon, and somatostatin.	Timper et al., 2006 [38]
		Total: 3 days		

Stage 1: chemically defined medium (CDM): 50% ICDM + 50% F12 NUT-MIX, insulin-transferrin-selenium-A, monothioglycerol, albumin fraction V, and *β*-mercaptoethanol	Human embryonic stem cells	Stage 1: 2 days	Activin A induced definitive endoderm differentiation from human embryonic stem cells with detection of the expression of definitive endoderm markers Sox17 and Brachyury. Retinoic acid promoted pancreatic differentiation, indicated by the expression of early pancreatic transcription factors Pdx1 and Hlxb9; bFGF and nicotinamide helped the differentiated cells to express islet specific markers such as C-peptide, insulin, glucagon, and glut2. Differentiated cells were able to secrete insulin in response to glucose stimulation *in vitro*.	
Stage 2: CDM, activin A		Stage 2: 4 days		
Stage 3: induced cells transferred into CDM with retinoic acid		Stage 3: 4 days	Transplanted cells in streptozotocin-induced nude mice survived and maintained expression of beta cell marker genes (C-peptide, Pdx-1, glucokinase, Nkx6.1, IAPP, Pax6, and Tcf1). 30% of mice showed restoration of stable euglycaemia for more than 6 weeks	Jiang et al., 2007 [27]
Stage 4: maturation medium (DMEM/F12, insulin-transferrin-selenium-A, albumin fraction V, bFGF		Stage 4: 3 days		
Stage 5: addition of nicotinamide, removal of bFGF				
		Stage 5: 5 days		
		Total: 18 days		

Stage 1: serum free H-DMEM medium, *β*-mercaptoethanol	Human bone marrow-derived mesenchymal stem cells from diabetic patients	Stage 1: 2 days	Transdifferentiated cells tested positive for dithizone and immunohistochemistry for insulin, PDX-1, Neurogenin3, Pax4, insulin, glucagon by RT-PCR; they also responded to glucose stimulation *in vitro *	
Stage 2: DMEM medium, bFGF, EGF, B27, and non-essential amino acids		Stage 2: 8 days		Sun et al., 2007 [35]
Stage 3: DMEM medium, betacellulin, activin A, nicotinamide, B27		Stage 3: 8 days		
		Total: 18 days		

Stage 1: DMEM/F12 medium, 15% FCS, progesterone, putrescine, laminin, insulin, sodium selenite, nicotinamide, transferring, and fibronectin	Human umbilical cord blood-derived stem cells with embryonic stem cell phenotype	Stage 1: 24 hours	Insulin-producing islet-like structures that co-expressed insulin and C-peptide were observed	Sun et al., 2007 [46]
Stage 2: H-DMEM medium, 15% FCS, progesterone, putrescine, laminin, insulin, sodium selnite, nicotinamide, transferring, and fibronectin		Stage 2: pancreatic islet-like structure started to appear after 5–7 days of induction		
		Total: up to 7 days		

Stage 1: H-DMEM medium, 5% FBS	Bone-marrow mesenchymal stem cells from Sprague-Dawly rats	Stage 1: 14 days	Islets like clusters were observed at the end of induction. Electron microscopy showed increased cytoplasmic secretory granules in differentiated cells. Differentiated cells insulin secretion increased by 1.5-fold after glucose challenge *in vitro*. After transplantation of islet-like clusters in diabetic rats, islet-like cells expressed islet hormones and lowered glucose levels of diabetic rats during day 6 to day 20	
Stage 2: addition of nicotinamide to the above medium		Stage 2: 7 days		Wu et al., 2007 [47]
Stage 3: addition of exendin-4		Stage 3: 7 days		
		Total: 28 days		

Stage 1:serum free DMEM medium, DMSO	Adult bone marrow stem cells from the long bones of rats	Stage 1: 3 days	Observation of islet-like clusters stained positive for dithizone. Differentiated cells showed expression of insulin and endocrine-specific genes. Differentiated cells showed *in vitro* glucose secretion in a dose-response manner when challenged with increasing glucose concentrations	
Stage 2: H-DMEM medium, 10% FBS, pancreatic extract		Stage 2: 7 days		Gabr et al., 2008 [36]
Stage 3: L-DMEM medium, 5% FBS, nicotinamide, and exendin-4		Stage 3: 7 days		
		Total: 17 days		

Stage 1: H-DMEM medium, 10% FBS, retinoic acid (24 hours), H-DMEM medium, 10% FBS (2 days)	Human umbilical cord blood-derived mesenchymal stem cells	Stage 1: 3 days	Islet-like cell clusters appeared 9 days after pancreatic differentiation. Insulin-secreting cells accounted for approximately 25% of the induced cells. Induced cells expressed islet-related genes and hormones but were not responsive to glucose challenge. Induced cells that were cultured without extracellular matrix gel failed to form clusters, and functional islet proteins were absent	
Stage 2: L-DMEM medium, 10% FBS, nicotinamide, EGF seeded in wells with extracellular matrix gel		Stage 2: 6 days		Gao et al., 2008 [37]
Stage 3: L-DMEM medium, 10% FBS, exendin		Stage 3: 6 days		
		Total: 15 days		

Stage 1: expansion of human umbilical cord mesenchymal cells in neuronal conditioned medium	Mesenchymal stem cells in Wharton's jelly of human umbilical cord	Stage 1: 7 days	Transdifferentiated cells formed islet-like clusters. RT-PCR showed expression of Pdx1, Hlxb9, Nkx2.2, Nkx6.1, and Glut-2. Islet-like clusters capable of producing insulin both *in vitro and in vivo *	
Stage 2: generation of nestin positive cells in DMEM/F12 medium, 2% FBS, nicotinamide, and B27		Stage 2: 7 days		Chao et al., 2008 [48]
Stage 3: differentiation of premature clusters in DMEM/F12 medium, 2% FBS, nicotinamide, B27, and stem cell conditioned medium		Stage 3: 14 days		
Stage 4: maturation of insulin-secreting cells				
		Total: 28 days (excluding stage 4)		

FBS: foetal bovine serum, FCS: foetal calf serum, H-DMEM: high-glucose DMEM, L-DMEM: low-glucose DMEM.

**Table 3:** Extrinsic factors involved in insulin-producing cell generation.

Extrinsic factor	Effect	Author
bFGF	Beta-cell differentiation	Dalvi et al., 2009 [49]
	Potent chemoattractant. May be useful in cluster formation	Hardikar et al., 2003 [50]

EGF	High concentration may be inhibitory to beta-cell differentiation	Cras-Méneur et al. 2001 [51], Dalvi et al., 2009 [49]

bFGF and EGF	Differentiation of embryonic stem cells into IPCs	Lumelsky et al., 2001 [52]
	Differentiation of human bone marrow-derived mesenchymal stem cells into IPCs	Sun et al., 2007 [35]

Betacellulin	Formation of islet-like clusters	Demeterco et al., 2000 [53]
	Induction of beta cell differentiation	

Activin A	Increase in insulin content	Demeterco et al., 2000 [53]

Betacellulin and activin A	Differentiation of pancreatic acinar AR42J cells into IPCs	Mashima et al., 1996 [54]
	Combined effect may be weaker than that of either factor alone	Demeterco et al., 2000 [53]
	Differentiation of human liver-derived insulin-producing cells toward the beta-cell phenotype	Zalzman et al., 2005 [40]
	Differentiation of human bone marrow-derived mesenchymal stem cells into IPCs	Sun et al., 2007 [35]

Nicotinamide	Differentiation of stem cells of various origins into IPCs	Chen et al., 2004 [44], Segev et al., 2004 [3], Tang et al., 2004 [45], Sun et al., 2007 [35], Sun et al., 2007 [46], Wu et al., 2007 [47], Chao et al., 2008 [48], Gabr et al., 2008 [36], Gao et al., 2008 [37]
	Increase in insulin content, DNA content, expression of insulin, glucagon, and somatostatin genes	Otonkoski et al., 1993 [55]

Exendin-4	Differentiation of murine bone marrow stem cells into IPCs	Tang et al., 2004 [45]
	Formation of insulin-expressing cells generated from adipose tissue-derived mesenchymal stem cells	Timper et al., 2006 [38]
	Differentiation of rat bone marrow-derived mesenchymal stem cells into IPCs	Wu et al., 2007 [47]
	Differentiation of rat bone marrow-derived stem cells into IPCs	Gabr et al., 2008 [36]
	Increase in insulin release by IPCs generated from mouse embryonic stem cells	Li et al., 2010 [56]

Hepatocyte growth factor	Differentiation of pancreatic acinar cells into IPCs	Mashima et al., 1996 [54]
	Increase in the number of IPCs in cultured human islets	Otonkoski et al., 1996 [57]

Gastrin	Stimulation of islet differentiation and islet growth	Wang et al., 1993 [58], Wang et al., 1997 [59]

Glucose	Low concentration (5 mM) increased insulin content.High concentrations (20–30 mM) increased beta cell replication	Bonner-Weir et al., 1989 [60]
